# A multilayer dynamic perturbation analysis method for predicting ligand–protein interactions

**DOI:** 10.1186/s12859-022-04995-2

**Published:** 2022-11-02

**Authors:** Lin Gu, Bin Li, Dengming Ming

**Affiliations:** College of Biotechnology and Pharmaceutical Engineering, Nanjing Tech University, Biotech Building Room B1-404, 30 South Puzhu Road, Jiangbei New District, Nanjing City, 211816 Jiangsu People’s Republic of China

**Keywords:** Ligand–protein interaction, Ligand spatial extension, Multilayer, Dynamics perturbation analysis, DPA

## Abstract

**Background:**

Ligand–protein interactions play a key role in defining protein function, and detecting natural ligands for a given protein is thus a very important bioengineering task. In particular, with the rapid development of AI-based structure prediction algorithms, batch structural models with high reliability and accuracy can be obtained at low cost, giving rise to the urgent requirement for the prediction of natural ligands based on protein structures. In recent years, although several structure-based methods have been developed to predict ligand-binding pockets and ligand-binding sites, accurate and rapid methods are still lacking, especially for the prediction of ligand-binding regions and the spatial extension of ligands in the pockets.

**Results:**

In this paper, we proposed a multilayer dynamics perturbation analysis (MDPA) method for predicting ligand-binding regions based solely on protein structure, which is an extended version of our previously developed fast dynamic perturbation analysis (FDPA) method. In MDPA/FDPA, ligand binding tends to occur in regions that cause large changes in protein conformational dynamics. MDPA, examined using a standard validation dataset of ligand-protein complexes, yielded an averaged ligand-binding site prediction Matthews coefficient of 0.40, with a prediction precision of at least 50% for 71% of the cases. In particular, for 80% of the cases, the predicted ligand-binding region overlaps the natural ligand by at least 50%. The method was also compared with other state-of-the-art structure-based methods.

**Conclusions:**

MDPA is a structure-based method to detect ligand-binding regions on protein surface. Our calculations suggested that a range of spaces inside the protein pockets has subtle interactions with the protein, which can significantly impact on the overall dynamics of the protein. This work provides a valuable tool as a starting point upon which further docking and analysis methods can be used for natural ligand detection in protein functional annotation. The source code of MDPA method is freely available at: https://github.com/mingdengming/mdpa.

**Supplementary Information:**

The online version contains supplementary material available at 10.1186/s12859-022-04995-2.

## Introduction

Proteins are ubiquitous in the cellular environment. They are not only the main components of cells but also the vital workforce to maintain the biochemical function of life. Proteins achieve their functions by interacting with a variety of molecules in cells, including some macromolecules (DNA, RNA, membranes) and small molecules (catalytic substrates, nucleotides, peptides, and artificial chemicals, often called ligands) [1, 2]. These interactions usually occur at several key amino acids in proteins, known as ligand-binding sites(LBSs), so the identification and characterization of protein LBSs and their associated ligands is key to understanding enzyme catalytic mechanism, disease pathogenesis and provide indispensable knowledge for enzyme engineering and drug design [3–6]. Furthermore, identifying protein LBSs is typically an indispensable starting step in virtual screening calculations, such as finding inhibitors or screening enzymes that bind to specific substrates [7].

Over the years, biological experiments have provided a wealth of protein structural data spanning all domains of life, which are available from the Protein Data Bank (PDB [8]), providing valuable, highly accurate protein LBS data. The rapid accumulation of experimental protein LBS data has been compiled into different databases, such as PoSSuM [9], BioLip [10], PLIC [11], sc-PDB [12], PDBbind [13], eModel-DBD [14], MOAD [15], etc., for different applications, giving birth to many computational methods for detecting and analyzing protein LBSs [16]. In recent years, not only has the number of known protein structures characterized experimentally increased rapidly, but advanced protein structure prediction programs are giving an ever-increasing number of new structures with reliable accuracy at an unprecedented rate [17, 18], which poses a great new challenge for the computational characterization of protein LBSs. At present, a variety of computational methods have been developed in the field of protein LBS prediction. According to their main algorithms, they can be divided into geometric-, energy-, template-/consensus-, knowledge-based methods and machine-learning methods; see references [19, 20] for details. Here, we mainly limited ourselves to a few geometric- or structure-based dynamic approaches that are closely related to this study, with particular emphasis on those that are freely available and freestanding.

As an early geometric method, POCKET [21] and the derivative LigSite [22] used geometric grids to explore protein-solvent-protein events and predicted those grids that begin and end with “protein” and into which solvents can be inserted as pockets. SURFNET [23] and PASS [24] used a probing ball randomly rotating on protein surface to search candidate pockets. FPOCKET [25] is a stand-alone geometric method that produces grid points based on Voronoi tessellation. For a given protein, FPOCKET usually generates many pockets which cover most protein LBSs, but they are not always in the top pocket. However, due to its easy-to-use, stand-alone and fast, it is very popular in the field and has been used as a built-in component by other programs, such as P2Rank [19]. Other methods introduce energy calculation on the basis of geometric representation. Q-SiteFinder [26] places methyl probes (-CH3) at grid points, calculates van der Waals interaction energies between probe atoms and neighboring protein atoms, and then clusters low-energy probes into candidate pockets as predictions. SiteHound [27] calculates the interaction forces between grid points and the protein, and clusters those points with higher interaction energies as candidate pockets. Then comes the consensus method MetaPocket 2.0 [28], a popular web server that combines the results produced by the above mentioned methods, including LigSite, PASS, Q-SiteFinder, Surfnet, FPOCKET and other three methods to improve the overall prediction success rate. Other consensus methods include COACH [29], G-LoSA [30], Libra [31], bSiteFinder [32], eMatchSite [33], etc. The template-based or consensus methods were reported to be the most successful and useful LBS prediction tools in CASP [34], which computationally determine conserved sites for certain protein families that bind target ligands based on sequence alignments [35]. Machine learning methods classify LBSs or protein functional residues based on structural, physico chemical features of biomolecular sequences/structures in the training sets [36–41]. In recent years, just like their development in protein structure prediction, machine learning and deep learning methods have also developed the fastest in the field of ligand-binding pocket and LBS prediction, as driven by the rapid accumulation of large amounts of experimental data [19, 42–45].

We have developed a template-free, structure-based and stand-alone method to predict protein LBS based on the protein dynamics perturbation analysis (DPA, and its Fast version FDPA) [46, 47]. The algorithm is based on the observation that external interactions, such as ligand binding, tend to occur in protein regions where interactions cause large conformational distribution changes, as measured by the allosteric potential *D*_x_ [46], which is the Kullback–Leibler divergence between protein conformational distributions with and without interaction. The MSMS program [48] was used to generate a layer of test points with a 1.5 Å radius on the surface of the protein, and those points with higher *D*_x_ values were selected and clustered to form prediction regions. In DPA, proteins were treated as some elastic network structures, and the external interactions between test points and neighboring protein atoms were simulated by connecting springs. The method was evaluated using 305 proteins in GOLD test set [49] of the time, and DPA yielded 287 protein-pocket predictions (rate of 94%), of which 250 predictions (rate of 87%) gave at least one correct LBS, while FDPA predicted 267 (rate of 86%) and 251 (rate of 94%), respectively. FDPA costs about 3 s to make a prediction for a protein of 130 aa with 2.2 GHz Intel Core i7 processor. We noted that since the DPA/FDPA algorithms only use a set of two-dimensional test points that are closely parallel to the protein surface (with an average distance of 1.5 Å from the protein surface), they cannot give the spatial distribution of target compounds (ligand or substrate) inside the pocket. However, in addition to LBSs and pockets, the spatial extensions of ligands within pockets are also important in practice [50] and their predictions have also received increasing attention over the years [51–55].

In this study, we presented a new version of DPA, called the multilayer dynamics perturbation analysis (MDPA), to predict both the protein LBSs and the spatial extension of ligands within the predicted pocket. The method was evaluated with a popular CCDC/Astex dataset for ligand-binding site prediction [56] and compared with the popular algorithms FPOCK [25] and CAVIAR [57]. The output of the algorithm might be used as input parameters for docking programs such as AutoDock [58], Vina [59], and others, and may also help optimize the conformation of the target ligand.

## Methods

### Implementation of MDPA

MDPA is an extension of the previously developed fast DPA or FDPA method. See the original work[47] for details of the FDPA. Here, only the principle and calculation process are outlined, with special emphasis on the extension of the method. This method is based on the observation that interactions introduced at LBSs tend to cause large perturbations to the protein conformational distribution, thereby making the protein activity susceptible to modulation by external molecular interactions at particular LBSs[46]. The conformational distribution of a protein containing N atoms, $P\left(\mathbf{X}\right)\propto exp(-U\left(\mathbf{X}\right)/{k}_{B}T)$, is a probability function of the protein configuration $\mathbf{X}=(\overrightarrow{{\mathbf{r}}_{1}},\overrightarrow{{\mathbf{r}}_{2}},\cdots ,\overrightarrow{{\mathbf{r}}_{{\varvec{N}}}})$, where $\overrightarrow{{\mathbf{r}}_{\mathbf{j}}}$ is the coordinate of *j*th atom. In DPA/FDPA, the potential energy $U(\mathbf{X})$ was modeled in harmonic approximation as a quadratic function $U(\mathbf{X})=E(\Delta \mathbf{X})=\frac{1}{2}{\Delta {\varvec{X}}}^{T}\mathbf{H}\Delta \mathbf{X}$**,** where $\Delta \mathbf{X}=\mathbf{X}-{\mathbf{X}}_{0}$ measures the protein deformation from its equilibrium configuration ${\mathbf{X}}_{0}$, and $\mathbf{H}$, called the Hessian matrix, is the second partial mass-weighted derivative matrix of the potential energy $U$ evaluated at ${\mathbf{X}}_{0}$ with respect to local coordinate changes. Harmonic approximation of the potential energy function *U* is usually valid because, under normal conditions, biomacromolecules must keep their structural fluctuations within a narrow range to perform their function correctly [60].

The key step of DPA/FDPA was to introduce external test interactions at random position “a” on the protein surface. For a given protein conformation $\mathbf{X}$, the algorithm determined the new configuration distribution ${P}^{({\varvec{a}})}(\mathbf{X})$ with the external interaction at “a” and measured its difference from unperturbed distribution $P(\mathbf{X})$ based on the Kullback–Leibler divergence, giving the *D*-value: ${D}^{(a)}=\int d\mathbf{X}\mathbf{l}\mathbf{n}\left(\frac{{P}^{({\varvec{a}})}(\mathbf{X})}{P(\mathbf{X})}\right){P}^{({\varvec{a}})}(\mathbf{X})$ [61]. In practice, the configuration distribution $P(\mathbf{X})$ was approximated by Boltzmann distribution with the potential energy $U(\mathbf{X})$ modeled using the ${C}_{\alpha }$-based elastic network model [62, 63], and an analytic solution of *D*^(*a*)^ was derived (depending on the protein equilibrium conformation ${\mathbf{X}}_{0}$). In DPA/FDPA, a set of random test points, also called surface points, denoted as ${\mathbf{L}}_{1}=\left\{{a}_{j}^{(1)},j=\mathrm{1,2},...,{S}_{1}\right\},$ was generated by the MSMS program [48], and those points with higher *D*-value (${D}^{({a}^{(1)})})$ were screened out and grouped into small clusters ${O}^{(1)},{P}^{(1)},...$, by using the OPTICS[64] clustering algorithm. Each cluster represented a predicted pocket, and protein residues near the cluster were then predicted as LBSs.

In DPA/FDPA, the set of test points generated by MSMS usually forms a closed surface parallel to the protein surface, which in turn makes the predicted pocket consisting of selected surface points very close (1.5 Å) to protein residues in any case. This often differs from what we observed when small molecules bind to proteins. In fact, except for a few marginal atoms, the entire small molecule tends to be located in the center of the binding pocket, some distance from the surface residues. Considering the importance of this ligand spatial extension in pockets, here we introduce a simple generalization of FDPA, called multilayer DPA or MDPA, for predicting protein LBSs and ligand spatial extension in pockets. MDPA treats the surface points ${\mathbf{L}}_{1}$ as a layer of virtual alpha-carbon atoms covering the protein structure, and then used MSMS program to generate a new set of surface points ${\mathbf{L}}_{2}=\left\{{a}_{j}^{(2)},j=\mathrm{1,2},...,{S}_{2}\right\}$ outside ${\mathbf{L}}_{1}$, using complex structure composed of the protein and ${\mathbf{L}}_{1}$. The *D*-values ${D}^{({a}^{(2)})}$ for ${\mathbf{L}}_{2}$ surface points $a_{j}^{\left( 2 \right)} \;^{\prime } s$ are determined using a similar analytic solution formula in FDPA, and those with higher *D*-values were screened out, and grouped into clusters ${O}^{(2)},{P}^{(2)},...$. Compared to pockets $\left\{{O}^{(1)},{P}^{(1)},...\right\}$ predicted based on layer 1 surface points, pockets $\left\{{O}^{(2)},{P}^{(2)},...\right\}$ are predicted to be farther from the protein surface, with an average distance of twice the average distance from ${\mathbf{L}}_{1}$ to the protein surface, i.e., twice the diameter of the probe sphere. Repeating this process, the third layer of surface points ${\mathbf{L}}_{3}$ was built on top of ${\mathbf{L}}_{2}$, giving predicted pockets $\left\{{O}^{(3)},{P}^{(3)},...\right\}$. Higher-layered pockets can also be generated in this way. In this study, only the pockets generated by the first three layers of test points were used, which was made after considering the balance between the prediction accuracy requirement and the computational load. Finally, the predicted pockets of all layers are collected, merged, and reclustered according to their spatial connectivity to obtain the final pocket predictions: ${O}_{M}, {P}_{M}$… Therefore, in many cases, a final predicted pocket, say ${O}_{M}$, may be the result of connection of pockets from multiple layers such that, say ${O}_{M}= {O}^{(1)}+{O}^{(2)}+{P}^{(3)}+...$, and each pocket was re-ranked according to the averaged D-value of test points in it. The prediction of protein LBSs was straightforward by simply finding amino acids whose Cα atom is within a distance of 6 Å from a point in the rank-1 predicted cluster (denoted as cluster ${O}_{m}$). LBSs predicted by lower-ranked pockets, if any, were also analyzed for comparison. The predicted clusters and protein structures were visualized by PyMOL (The PyMOL Molecular Graphics System, Version 2.5.2, Schrödinger, LLC.).

### Validation dataset

To verify the accuracy of the algorithm in predicting ligand-binding pockets, the CCDC/Astex dataset of 85 protein-ligand complexes were used in this study [56]. The dataset consists of a diverse, high-quality test set originally developed to evaluate protein-ligand docking programs. Most structures in the dataset have little global conformational similarity, but some of them are selected from several major protein families, including 11 kinases, 9 nuclear receptors, 5 serine proteases, and 3 members of the phosphodiesterase family. This dataset was originally based on the statistical analysis and classification of all protein-ligand complexes in the PDB database. Starting from the sequence of protein, the structure of the ligand, and the structure of the complex, the clustering of proteins with similar sequences was used to display as many different proteins as possible. Characterization of the complexes was accomplished by extracting the structure of the ligand, based on the number of heavy atoms and rotatable bonds in the ligand, and determining whether it has therapeutic or agrochemical use in the complex. 90% of these complexes were related protein targets, contain similar drug complexes, and contain high-quality experimental structures that facilitate the evaluation of the ligand binding modes.

### Validation of prediction LBSs and ligand spatial extension inside the binding pocket

To evaluate the prediction accuracy, we selected the set of protein residues ${R}_{P}$ whose Cα atoms were within 6 Å of any point in the rank-1 cluster ${O}_{m}$ and defined ${R}_{P}$ as the prediction LBSs. The prediction was compared with the set of LBSs ${R}_{L}$ found in the ligand-protein complex as collected from the SITE sections of the examined PDB files. The intersection of *R*_p_ and *R*_L_ defined a set of residues ${R}_{P\cap L}={R}_{P}\cap {R}_{L}$, recoding residues found in both the prediction and experimental determined LBSs. The overlap of the prediction with the experiment LBSs was then evaluated with the conventional prediction precision and recall: Precision = ${R}_{P\cap L}/{R}_{P}$, Recall = ${R}_{P\cap L}/{R}_{L}$.

Compared with DPA/FDPA, the new output of MDPA was a prediction of the spatial extension of ligands inside the predicted binding pocket. To evaluate the quality of predictions, we defined an evaluation strategy similar to evaluating LBS predictions. Let *S*_L_ be the set of atoms of a ligand found in the complex, and *S*_*l*_ be the subset of *S*_L_ whose elements are ligand atoms located within 3.5 Å of any test point of the predicted pocket ${O}_{M}$. Let ${O}_{m}$ be the subset of ${O}_{M}$, whose elements are test points located within 3.5 Å of any ligand atom. The overlap of the prediction with the spatial extension of the experimental ligand within the binding pocket was then assessed using precision and recall as follows: Precision = ${O}_{m}/ {O}_{M}$, and Recall = ${S}_{l}/ {S}_{L}$.

### Compared with FPOCKET method

As a fast geometric stand-alone method, FPOCKET has become one of the most popular tools for analyzing protein structural pockets in recent years. The method is based on the determination of alpha spheres by Voronoi tessellation [25]. An alpha sphere is a sphere that contacts four atoms at the protein boundary, and the radius of the alpha sphere reflects the local curvature defined by the four atoms. For a protein, very small spheres are located inside the protein, large spheres are located outside the protein, and cracks and cavities correspond to spheres of intermediate radii. Therefore, spheres of different radius sizes are filtered to solve the pocket detection problem. The algorithm first inputs the PDB structure, then returns the pre-filtered set of spheres, and finally identifies groups of closely connected alpha spheres, returns the pocket information, and scores each pocket. FPOCKET typically gives a large number of predicted pockets, covering most known LBSs and ligand molecules, however, LBSs and ligands are not always associated with the top predicted pockets. For quantitative comparisons, similar precision and recall are also defined to evaluate the predictions of FPOCKET's top-ranked pockets, where test points are replaced with alpha spheres.

## Results

### MDPA detects regions causing large perturbation to protein dynamics

**Fig. 1:**
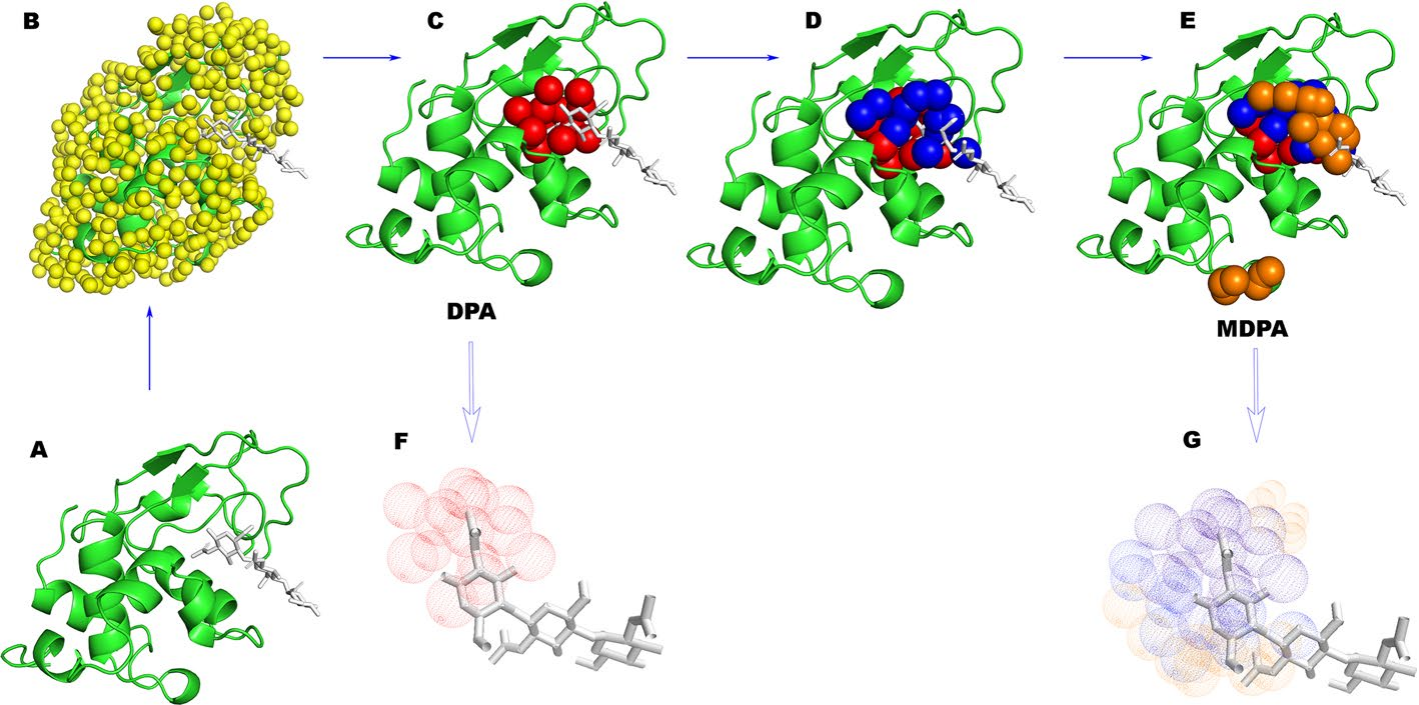
Multilayer Dynamics Perturbation Analysis (MDPA). **A**, **B** Test points (yellow) are created on the surface of the turkey egg-white lysozyme (PDB code: 1JEF [65], green cartoon). **C**, **F** DPA finds predicted regions (red) from first layer surface points, which are compared to the bound ligand. **C**, **D**, **E** MDPA finds predicted regions (red, dark blue, orange) from three layers of surface points and forms new predicted clusters, which are compared to the bound ligands(G)

The lysozyme from turkey egg-white (PDB code: 1JEF [65]) has been used as an example to illustrate the performance of DPA. Like many other lysozymes from different organisms, this protein consists of two domains connected by a helix linker, with its substrate polysaccharide bound deep in between the two lobes (Fig. 1A). Experimental data and simulation calculations show that the enzyme is constantly switching between open and fully closed conformations by adjusting the distance between the two lobes [66, 67]. As mentioned above, from random protein surface points, DPA selects a subset of surface points that most perturb the protein conformational distribution, which are then predicted to be associated with the binding site for protein substrates (Fig. 1). In the case of lysozyme, the interactions introduced at those surface points predicted by DPA should intuitively have the highest efficiency in blocking the key intrinsic opening/closing motion of the enzyme. The original DPA algorithm only considers the first layer (~ 1.5 Å from the protein surface) surface points (Fig. 1B), and it has the obvious disadvantage that it provides little information about the position and spatial extension of the substrate in the binding pocket (Fig. 1C, F). In MDPA, multiple layers of surface points are created on the protein surface, each layer is added to the top of the previous layer, then a subset of surface points with high DPA values are selected for each layer, and finally, all selected surface points are collected and clustered to form predicted binding region (Fig. 1C,D,E,G). For turkey egg-white lysozyme, both DPA and MDPA predicted the same ligand binding sites, namely D52, Q57, I58, N59, W62, W63, A107, and W108, giving a recall rate of 62% and an accuracy rate of 83% compared to the sites listed in the SITE entries of the PDB file. Compared to DPA (Additional file 1: Table S1), since it uses multiple layers of surface points, the main improvement of MDPA lies in the overlap between the predicted spatial region and the physical occupancy of the bound ligand, from 67% in DPA increased to 100% in MDPA, with a slight sacrifice in accuracy from 87 to 82%.

**Fig. 2:**
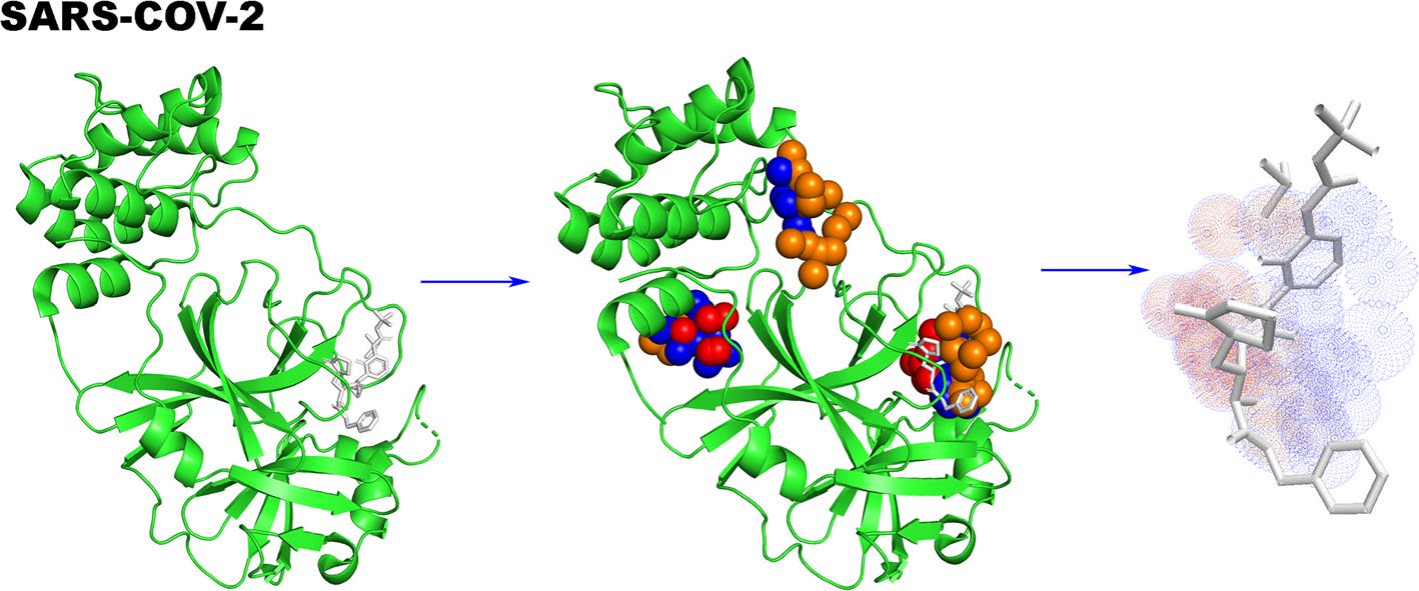
Prediction of ligand-binding regions in the multidomain structure of the SARS-CoV-2 main protease. The predicted rank-1 binding region locates between domain I and II, the 2nd and 3rd predicted regions are between domains II and III

As a second example, we applied MDPA to detect the ligand-binding pocket in the main protease of the recent global pandemic coronavirus. The COVID-19 caused by the novel coronavirus SARS-CoV-2 virus, coupled with the lack of targeted drugs, urgently needs to find new antiviral drugs. The main protease (M^pro^) of coronaviruses has been identified as a key drug target for the development of inhibitors to block viral RNA protein processing. The overall structure of the protease (PDB code 6Y2F[68]) can be divided into 3 consecutive and mutually contacting domains (domain I, II, and III) corresponding to residues 10 to 99, 100 to 182, and 198 to 303, respectively. Figure 2 shows the 3 predicted inhibitor-binding regions in the structure given by MDPA, with the rank-1 predicted region located between domains I and II, and the 2nd and 3rd prediction regions between domains II and III. Compared to recently reported α-ketoamide inhibitors[68, 69], in which Cys145 mediates the nucleophilic reaction and Gly143 and Glu166 form hydrogen bonds with the inhibitor, the predicted rank-1 region perfectly matches where the α-ketoamide inhibitor binds within the pocket. The prediction identified important binding sites such as Glu166, His164, Cys145, Gly143, His41, and Arg188, which accounts for half of the listed ligand-binding residues. The predicted binding region overlapped with the α-ketoamide inhibitor with 89% accuracy and 81% recall.

### Evaluation of MDPA for prediction of ligand-binding sites

One of the key features of protein pockets is the distribution of ligand-binding sites within them, which largely determines protein function. To evaluate the ability of MDPA to predict ligand-binding sites, MDPA was applied to the protein structures of the CCDC/Astex dataset of 85 protein-ligand complexes. The residues identified by 3-layered MDPA predictions were then compared to the ligand-binding sites listed in the SITE records for each complex structure (Table 1). MDPA made at least one prediction ligand-binding region for 94% of the listed complexes in the dataset, with the exception of the four cases where the ligand-binding region was embedded inside the internal protein cavities. Compared to the listed ligand binding sites in the PDB entry, the predictions were at least 30% accurate in 65% of cases and at least 50% accurate in 25% of cases; the recall rate of residues is at least 30% in 87% of cases and at least 50% in 71% of cases.

**Table 1:** Validation of MDPA method using the CCDC/Astex dataset

Entry		Prediction	Ligand-binding Site			Ligand orientation	
PDB	Chain	Cluster	MCC	Precision	Recall	Precision	Recall
1g9v	A	O	0.45	0.32	0.86	0.76	0.98
1gkc	A	O	0.56	0.47	0.75	0.76	0.73
1gm8	B	R	0.63	1.00	0.40	1.00	0.71
1gpk	A	O	0.41	0.23	0.83	0.32	0.94
1hnn	A	O	0.48	0.34	0.84	0.49	1.00
1hp0	A	O	0.69	0.50	1.00	0.93	1.00
1hq2	A	O	0.51	0.34	0.92	0.69	1.00
1hvy	A	O	0.38	0.21	0.82	0.41	0.81
1hwi	AB	O	0.26	0.25	0.29	0.00	0.00
1hww	A	P	0.15	0.25	0.09	0.00	0.00
1ia1	A	O	0.49	0.35	0.80	0.58	1.00
1ig3^†^	B	O	0.00	0.00	0.00	0.00	0.00
1j3j	A	O	0.55	0.42	0.80	0.88	1.00
1jd0	A	O	0.47	0.32	0.78	0.49	1.00
1jje	A	O	0.49	0.33	0.82	0.61	1.00
1jla	A	O	0.22	0.13	0.44	0.14	0.33
1k3u	A	O	0.52	0.33	0.93	0.48	1.00
1ke5	A	O	0.14	0.18	0.17	0.78	0.13
1kzk	A	O	0.49	0.55	0.55	0.45	0.44
1l2s	A	O	0.41	0.21	0.88	0.38	1.00
1l7f	A	O	0.37	0.29	0.54	0.54	1.00
1lpz	B	O	0.60	0.62	0.62	0.87	0.88
1lrh	A	O	0.52	0.35	0.89	0.79	1.00
1m2z	A	O	0.17	0.22	0.18	0.00	0.00
1meh	A	O	0.46	0.60	0.38	0.67	0.30
1mmv	A	O	0.11	0.09	0.22	0.46	0.74
1mzc	B	O	0.07	0.08	0.10	0.00	0.00
1n1m^*^	A	O	0.00	0.00	0.00	0.00	0.00
1n2j	A	O	0.29	0.12	0.83	0.26	1.00
1n2v	A	O	0.50	0.30	0.89	0.62	1.00
1n46	A	O	0.01	0.08	0.06	0.00	0.00
1nav	A	O	0.48	0.45	0.60	1.00	0.87
1of1	A	O	0.82	0.78	0.88	1.00	1.00
1of6	A	O	0.66	0.71	0.62	0.40	0.62
1opk	A	O	0.07	0.05	0.17	0.00	0.00
1oq5	A	O	0.60	0.39	1.00	0.67	1.00
1owe	A	O	0.50	0.27	1.00	0.29	1.00
1oyt	H	O	0.51	0.43	0.69	0.73	0.97
1p2y^*^	A	O	0.00	0.00	0.00	0.00	0.00
1p62	B	O	0.48	0.36	0.73	0.88	1.00
1pmm	A	O	0.38	0.38	0.42	0.55	0.60
1q1g	A	O	0.44	0.31	0.73	0.58	1.00
1q41	A	O	0.06	0.07	0.12	0.50	0.29
1q4g	A	O	0.45	0.24	0.88	0.31	1.00
1r1h^*^	A	O	0.00	0.00	0.00	0.00	0.00
1r55	A	O	0.65	0.59	0.77	0.83	0.83
1r58	A	O	0.52	0.41	0.70	0.83	0.70
1r9o	A	O	0.26	0.13	0.62	0.10	0.39
1s19	A	O	0.52	0.33	0.92	0.98	0.97
1s3v	A	O	0.57	0.53	0.69	0.89	0.48
1sg0	AB	O	0.45	0.29	0.75	0.50	0.10
1sj0	A	O	0.49	0.50	0.54	0.82	0.33
1sq5	A	O	0.54	0.58	0.54	0.29	0.48
1sqn	A	O	0.21	0.17	0.38	0.00	0.00
1t40	A	O	0.48	0.42	0.62	0.59	0.96
1t46	A	O	0.39	0.36	0.50	0.34	0.51
1t9b	A	O	0.40	0.33	0.50	0.85	0.52
1tow	A	O	0.16	0.10	0.67	0.50	0.95
1tt1	A	O	0.69	0.78	0.64	1.00	0.73
1tz8	AB	O	0.72	0.23	0.57	0.22	0.50
1u1c	A	O	0.38	0.23	0.78	0.54	1.00
1u4d	A	O	0.47	0.28	0.89	0.47	0.94
1uml	A	O	0.63	0.41	1.00	0.74	1.00
1unl	A	O	0.28	0.28	0.36	0.87	0.54
1uou^*^	A	O	0.00	0.00	0.00	0.00	0.00
1v0p	A	O	0.53	0.71	0.42	1.00	0.33
1v48	A	P	0.65	0.69	0.64	0.77	0.91
1v4s	A	O	0.73	0.71	0.77	1.00	0.61
1vcj	A	O	0.35	0.33	0.42	0.85	1.00
1w1p	A	O	0.27	0.07	1.00	0.17	1.00
1w2g	A	O	0.29	0.11	1.00	0.07	1.00
1 × 8x	A	P	0.20	0.17	0.33	0.37	0.69
1xm6	A	O	0.47	0.31	0.8	0.58	1.00
1xoq	A	O	0.59	0.43	0.87	0.56	1.00
1xoz	A	O	0.36	0.25	0.6	0.39	0.66
1y6b	A	O	0.56	0.48	0.71	0.81	0.79
1ygc	H	O	0.47	0.50	0.50	0.96	0.89
1yqy	A	Q	0.3	0.43	0.23	0.44	0.43
1yv3	A	P	0.38	0.50	0.31	0.35	0.48
1yvf	A	P	0.31	0.10	1.00	0.16	1.00
1ywr	A	O	0.53	0.40	0.77	0.70	0.97
1z95	A	O	0.22	0.23	0.33	0.00	0.00
2bm2	A	P	0.48	0.56	0.45	0.25	0.03
2br1	A	O	0.39	0.30	0.58	0.40	0.45
2bsm	A	O	0.52	0.43	0.71	0.78	0.78

*cases where ligands are embedded in a cavity inside the proteins. "AB" represents the structure of the biological unit as a dimer

### Evaluation of MDPA for prediction of ligand spatial extension within the pocket

**Fig. 3:**
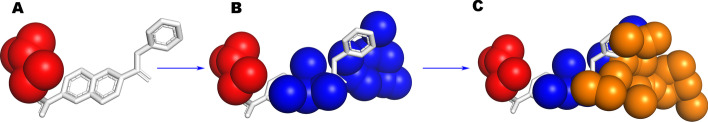
The predicted region grows layer-by-layer along the natural ligand in the pocket of human serine protease urokinase plasminogen activator (PDB code: 1OWE [70]). Layers 1 to 3 are colored red, blue, and orange, respectively

Compared with previous DPA studies, the development of the MDPA algorithm aims to improve the prediction of the spatial extension of ligands in protein pockets and provide useful information for subsequent ligand prediction and ligand-protein docking calculations. Any medium to large-sized ligand usually adopts a specific spatial distribution within the pocket, with parts of the ligand structure close to the protein and others away from the protein. Both DPA and MPA identify surface point clusters that impose high perturbation on the overall dynamics of the protein through interactions with neighboring protein amino acids, and according to this method, it is easier to detect interacting point sets closer to the protein surface. A key step in MDPA is to expand the collection of highly perturbing points at increasing distances from the protein surface in a layer-by-layer manner. Therefore, it would be interesting to verify the correlation between the direction of the predicted region increase and the spatial extension of ligands in the pocket. As an example, Fig. 3 shows that MPDA successfully computed the growth of the predicted region along the direction of the spatial distribution of the ligand 6-substituted 2-naphthamidine inhibitor in the human urokinase pocket (PDB code: 1OWE[70]). In this case, the predicted overlap of the binding region with the protein inhibitor increased from 30% in DPA to 100% in MDPA. To evaluate its ability to predict the spatial distribution of ligands in the pocket, MDPA was applied to the CCDC/Astex dataset of 85 protein-ligand complexes, and the overlap ratios of the predicted regions with the natural ligands found in the complexes were calculated (Table 1). Calculations showed that the predicted binding region of MDPA overlapped with the natural ligand in 82% of the complexes. Among them, the prediction accuracy is at least 30% in 87% of cases and at least 50% in 62% of cases, with the recall rate at least 30% in 95% of cases and at least 50% in 80% of cases.

## Discussions

### Compared to FPOCKET and CAVIAR

**Fig. 4:**
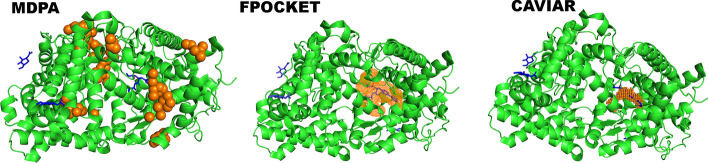
Pocket prediction of human neprilysin (PDB code: 1R1H) calculated by MDPA, FPOCKET and CAVIAR. The protein in the complex structure is represented by green cartoon, the ligand in blue sticks. Predictions of MDPA and CAVIAR are shown as spheres, and that of FPOCKET as mesh

For comparison, we selected two free, standalone protein pocket prediction programs, FPOCKET [25] and CAVIAR, to examine MDPA by comparing their predictions of protein pockets of the complexes in the dataset (Additional file 1: Tables S2 and S3). FPOCKET identifies cavities through spherical probes, while CAVIAR detects pockets according to the grid algorithm, so most binding sites can be presented by a large number of pockets. Although calculations show that FPOCKET and CAVIAR can effectively identify the ligand-binding pockets of most proteins, the overlap rate between the predicted point sets and the bound ligand of the complexes is relatively small, with an average of < 10%, which provides little information about the ligand-binding position and spatial extension inside the pockets. Compared with FPOCKET and CAVIAR (Table 2), MDPA has higher accuracy in predicting binding sites (60%) and ligand spatial extension (71%), avoiding redundancy. We also noted that if the pocket is completely embedded inside the protein, MDPA does not generate any surface points in this region with the MSMS program, and its predicted binding region has zero overlap with the corresponding ligand (Fig. 4, PDB code: 1R1H[71]). Therefore, MDPA does not work for pockets buried in the protein core, whereas both FPOCKET and CAVIAR may correctly predict the embedded binding pocket. In this sense, MDPA provides a better description of the ligand-binding pose in pockets located on the protein’s outer surface, and this information is expected to be useful for further molecular docking calculations and other ligand–protein interaction characterizations.

**Table 2:** MDPA is compared with FPOCKET and CAVIAR for the prediction of binding pockets of the structures in the CCDC/Astex dataset

Method	Ligand-binding Site Prediction		Ligand overlap	
	percentage with precision > 30%	percentage with recall > 30%	percentage with precision > 30%	percentage with recall > 30%
MDPA	60%	80%	71%	78%
FPOCKET	56%	85%	65%	81%
CAVIAR	–	–	70%	89%

### Combining deep-learning models to improve the prediction accuracy of ligand binding pockets

Data-driven methods have long been used to predict important ligand binding residues in proteins and physicochemical properties involving ligand-protein interactions [19]. Recent years have seen an increasing number of deep-learning models reported to predict ligand-binding pockets or ligand-binding sites [19, 42, 72–75]. For example, Zhang and colleagues [76] developed a free and standalone deep-learning model called DeepbindPoc, which uses a 3D convolutional neural network to rank the predicted pockets identified by FPOCKET to screen for the pockets most likely to bind natural ligands. An interesting feature of DeepbindPoc is that the algorithm uses the mol2vec tool to add information about natural ligands to the training dataset, so it has the advantage of ranking near-native pockets for proteins with unknown binding sites but whose ligands are known in advance, and it may also help predict natural ligands for a given protein. Combining DeepbindPoc with MDPA, DeepbindPoc helps reprioritize the predicted regions identified by MDPA by using natural ligands(Additional file 1: Table S4). Of the 85 complexes, the highest-scoring pockets given by DeepbindPoc were consistent with the rank-1 pockets predicted by MDPA in 51% of cases, while the remaining 48% were different. In the cases where the two do not match, there are pros and cons between MDPA prediction ranking and DeepbindPoc scoring. For example, in the case of the influenza virus neuraminidase-inhibitor complex (PDB code: 1L7F [77]), DeepbindPoc gave the highest score to the fourth-ranked MDPA pocket, which does bind the ligand BCX-1812, while the rank-1 MDPA pocket has no binding ligand. However, in most cases (72%), the pockets screened by DeepbindPoc score do not seem as reasonable as the top-ranked pockets predicted by MDPA. The DeepbindPoc scoring function provides some references for MDPA-predicted pockets that do not bind to natural ligands. Therefore, screening candidate ligands of these pockets using tools such as DeepbindPoc is worth further study.

### Effects of protein conformation changes on protein pocket detection

**Fig. 5:**
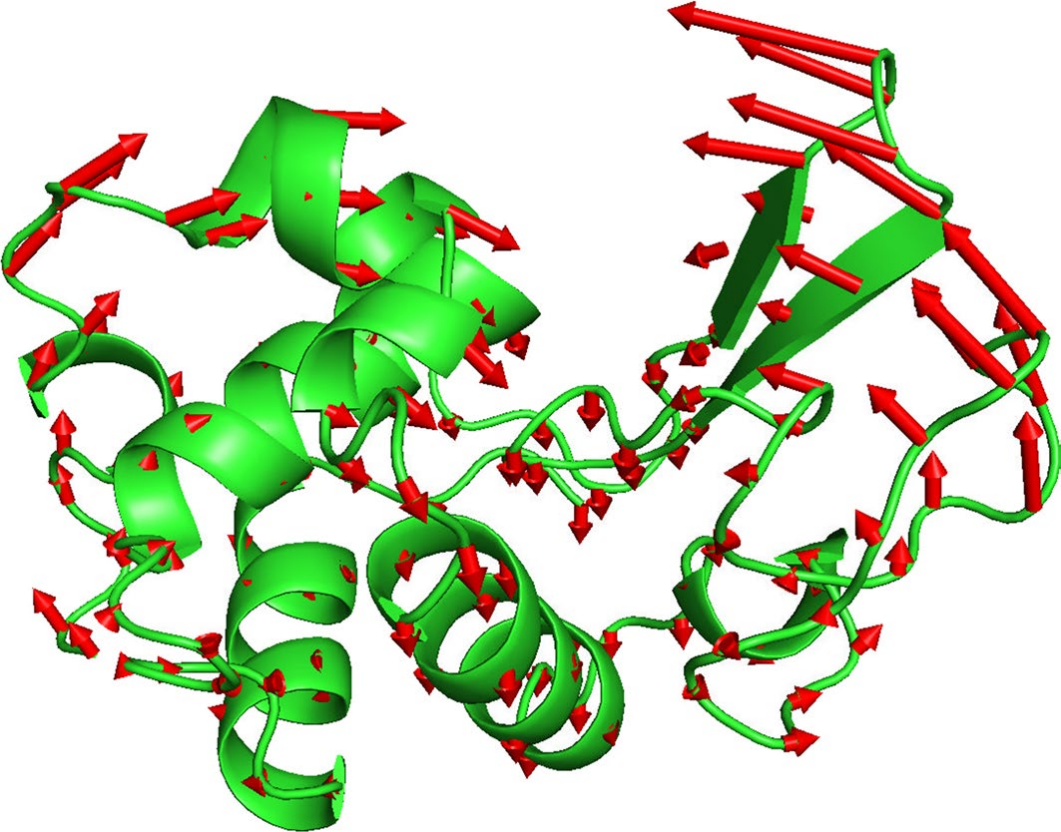
Changes in the shape of the protein pocket. Arrows illustrate the amplitude and direction of motion of the alpha carbon atoms when the human lysozyme (PDB code:1LZS) vibrates along the lowest frequency normal mode, a hinge motion involved in the opening and closing of the catalytic pocket. This conformational change has little effect on the MDPA prediction of the ligand-binding region of this protein

Just as flexible docking or flexible ligand recognition [78, 79] must take into account a certain degree of flexibility in the pocket side chain or backbone, it may be useful to examine the effect of protein conformational changes on the prediction of binding pockets. As an example, the human lysozyme was examined here, where structural changes in the binding pocket and different substrate binding modes have been characterized (PDB code: 1LZS [80]). The typical opening/closing conformational changes are simulated by the lowest frequency normal mode solved in MDPA (Fig. 5), where the entire structure rotates around a central axis connecting the two lobs.

Since no direct measurement data were available for the magnitude of the conformational change defined by the normal mode, a series of 12 conformations were generated, evenly distributed through a full cycle, with amplitude $A\mathrm{cos}(i\pi /12), i=\mathrm{1,2},\dots ,12$, where *A* is an arbitrary value. To determine the maximum possible value of *A*, the Procheck[81] program was used to examine the distribution of unreasonable dihedral angles in the resulting conformations. Specifically, the largest *A* value was chosen under the condition that the Procheck program reports that at least 90% of the dihedral angles are within a reasonable area. MDPA was then applied to each conformation, and the predictions were compared with ligands for which binding sites were found (Table 3). The pocket was given a variety of flexibility from open to closed conformations, which mimics the conformational sampling of a flexible pocket. In the absence of conformational changes, the ligand-binding site predictions achieved 20% accuracy and 100% recall, and the ligand-spatial extension prediction achieved 80% accuracy and 100% recall. For comparison, a large conformational change $A$ =*200* and a medium-sized conformational change $A=100$ were applied to the protein (Table 3). Calculations showed that the predictions given by MDPA vary slightly with conformational changes. When $A=100$, the precision rate of LBS prediction reaches 31% in conformation 4 and 8, and the prediction accuracy of ligand spatial extension increases to 86%. In contrast, for the conformations derived by setting $A$ =*200*, the highest ligand spatial extension prediction accuracy is only 83%. The calculations revealed that properly increasing the flexible deformation of protein conformation can improve the accuracy of ligand spatial extension prediction. This may be particularly useful for predicting ligand-binding regions within relatively large binding pockets.

**Table 3:** Comarison of MDPA predictions of human lysozyme (PDB code: 1LZS) undergoing different conformation changes by applying the lowest frequency normal mode motion with different amplitudes

Amplitude	Entry	Ligand-binding Site Prediction		Ligand Overlap	
		Precision	Recall	Precision	Recall
100	00	0.29	1.00	0.83	1.00
	01	0.27	1.00	0.75	1.00
	02	0.27	1.00	0.75	1.00
	03	0.29	1.00	0.80	1.00
	04	0.31	1.00	0.86	1.00
	05	0.31	1.00	0.77	1.00
	06	0.31	1.00	0.75	1.00
	07	0.31	1.00	0.75	1.00
	08	0.31	1.00	0.86	1.00
	09	0.29	1.00	0.86	1.00
	10	0.27	1.00	0.75	1.00
	11	0.27	1.00	0.75	1.00
200	00	0.31	1.00	0.80	1.00
	01	0.31	1.00	0.82	1.00
	02	0.29	1.00	0.83	1.00
	03	0.31	1.00	0.80	1.00
	04	0.33	1.00	0.75	1.00
	05	0.33	1.00	0.82	1.00
	06	0.33	1.00	0.80	1.00
	07	0.31	1.00	0.82	1.00
	08	0.31	1.00	0.75	1.00
	09	0.29	1.00	0.80	1.00
	10	0.29	1.00	0.83	1.00
	11	0.31	1.00	0.82	1.00

On the other hand, we also noticed that, in many cases, ligand binding might lead to protein conformation changes that are not well described by the low-frequency normal mode motions described above, such as induced-fit conformational changes that cause strong inelastic deformations. In this case, it might be difficult to make accurate LBS predictions using the apo protein structures. Therefore, comparing LBS predictions based on apo structures with those based on complex structures may be another topic worthy of further study.

## Conclusion

In this study, a multilayer dynamic perturbation analysis method (MDPA) was developed to predict ligand-binding pockets in proteins. This structural-based method is a direct improvement on our previously established method. The method has been validated using a standard ligand-protein complex dataset, with an LBS prediction accuracy of at least 30% in 65% of cases and recall of at least 30% in 87% of cases. One of the key features of MDPA is that MDPA is designed to predict the spatial extension of ligands within the binding pocket. The predicted binding region overlapped with the natural ligand in 82% of the complexes in the test dataset, with a prediction accuracy of at least 30% in 87% of the cases and a recall of at least 30% in 95% of cases, indicating that MDPA can give a reasonable prediction for protein pickets.

The combination of MDPA with deep learning methods based on ligand-protein interaction big data (such as DeepbindPoc) provides a good starting point for MDPA to study further biomolecular functions, including the prediction of natural ligands and their involved physicochemical interactions.

## Supplementary Information

**Additional file 1: Table S1.** Prediction results of DPA for protein structures in CCDC/Asterx dataset; **Table S2.** Prediction results of FPOCKET for protein structures in CCDC/Asterx dataset; **Table S3.** Prediction results of CAVIAR for protein structures in CCDC/Asterx dataset; **Table S4.** Selection of optimal pockets for protein structures in CCDC/Asterx dataset by combining DeepbindPoc; **Figure S1.** MDPA calculation results of SARS-COV-2.

## Data Availability

All data is provided; the MDPA program code is also uploaded to https://github.com/mingdengming/mdpa
