# Supplementary material for: A multilayer dynamic perturbation analysis method for predicting ligand–protein interactions
Source: BMC Bioinformatics. 2022 Nov 2;23:456. doi: 10.1186/s12859-022-04995-2 (PMC9628359; doi:10.1186/s12859-022-04995-2)
Supplement: Supplementary file 1 — Additional file 1: Table S1. Prediction results of DPA for protein structures in CCDC/Asterx dataset; Table S2. Prediction results of FPOCKET for protein structures in CCDC/Asterx dataset; Table S3. Prediction results of CAVIAR for protein structures in CCDC/Asterx dataset; Table S4. Selection of optimal pockets for protein structures in CCDC/Asterx dataset by combining DeepbindPoc; Figure S1. MDPA calculation results of SARS-COV-2. [file 12859_2022_4995_MOESM1_ESM.docx]

**Supporting information**

***Table S1. Prediction results of DPA for protein structures in CCDC/Asterx dataset***

| ***Entry*** | | | ***Site prediction*** | | | ***Ligand space distribution*** | |
| --- | --- | --- | --- | --- | --- | --- | --- |
|  |  |  |  |  |  |  |  |
| ***PDB*** | ***Chain*** | ***Cluster*** | ***MCC*** | ***Precision*** | ***Recall*** | ***Precision*** | ***Recall*** |
| 1g9v | A | O | 0.45 | 0.32 | 0.86 | 0.76 | 0.98 |
| 1gkc | A | O | 0.52 | 0.47 | 0.57 | 0.80 | 0.45 |
| 1hnn | A | O | 0.48 | 0.34 | 0.84 | 0.49 | 1.00 |
| 1hp0 | A | O | 0.69 | 0.50 | 1.00 | 0.93 | 1.00 |
| 1hvy | A | O | 0.30 | 0.19 | 0.64 | 0.37 | 0.44 |
| 1j3j | A | O | 0.45 | 0.45 | 0.50 | 1.00 | 1.00 |
| 1jd0 | A | O | 0.46 | 0.35 | 0.67 | 0.56 | 0.69 |
| 1jje | A | O | 0.58 | 0.45 | 0.82 | 0.82 | 0.80 |
| 1jla | A | O | 0.28 | 0.20 | 0.44 | 0.38 | 0.26 |
| 1k3u | A | O | 0.57 | 0.39 | 0.93 | 0.76 | 1.00 |
| 1kzk | A | O | 0.28 | 0.43 | 0.27 | 0.20 | 0.05 |
| 1l2s | A | O | 0.25 | 0.20 | 0.38 | 0.10 | 0.11 |
| 1lrh | A | O | 0.53 | 0.36 | 0.89 | 0.87 | 1.00 |
| 1m2z | A | O | 0.17 | 0.22 | 0.18 | 0.00 | 0.00 |
| 1mmv | A | O | 0.19 | 0.20 | 0.22 | 0.67 | 0.14 |
| 1n1m | A | O | 0.00 | 0.00 | 0.00 | 0.00 | 0.00 |
| 1n2j | A | O | 0.30 | 0.13 | 0.83 | 0.33 | 1.00 |
| 1n46 | A | O | 0.04 | 0.11 | 0.06 | 0.00 | 0.00 |
| 1of1 | A | O | 0.82 | 0.78 | 0.88 | 1.00 | 1.00 |
| 1of6 | A | O | 0.00 | 0.00 | 0.00 | 0.00 | 0.00 |
| 1pmm | A | O | 0.05 | 0.07 | 0.08 | 0.00 | 0.00 |
| 1q1g | A | O | 0.46 | 0.33 | 0.73 | 0.56 | 0.80 |
| 1q4g | A | O | 0.34 | 0.33 | 0.38 | 0.75 | 0.60 |
| 1q41 | A | O | 0.07 | 0.08 | 0.12 | 0.67 | 0.29 |
| 1sg0 | AB | O | 0.43 | 0.58 | 0.35 | 1.00 | 0.36 |
| 1sq5 | A | O | 0.30 | 0.40 | 0.25 | 0.25 | 0.33 |
| 1sqn | A | O | 0.24 | 0.20 | 0.38 | 0.00 | 0.00 |
| 1t9b | A | O | 0.27 | 0.15 | 0.50 | 0.00 | 0.00 |
| 1tt1 | A | O | 0.69 | 0.78 | 0.64 | 1.00 | 0.73 |
| 1tz8 | AB | O | 0.72 | 0.23 | 0.57 | 0.67 | 0.50 |
| 1u1c | A | O | 0.43 | 0.27 | 0.78 | 0.50 | 0.55 |
| 1u4d | A | O | 0.38 | 0.25 | 0.67 | 0.31 | 0.50 |
| 1unl | A | O | 0.34 | 0.38 | 0.36 | 0.80 | 0.19 |
| 1v0p | A | O | 0.36 | 0.36 | 0.42 | 0.46 | 0.10 |
| 1w1p | A | O | 0.21 | 0.09 | 0.50 | 0.17 | 0.50 |
| 1w2g | A | O | 0.32 | 0.18 | 0.75 | 0.80 | 1.00 |
| 1xm6 | A | O | 0.47 | 0.31 | 0.80 | 0.58 | 1.00 |
| 1xoq | A | O | 0.61 | 0.50 | 0.80 | 0.72 | 0.85 |
| 2bm2 | A | P | 0.48 | 0.56 | 0.45 | 0.25 | 0.03 |
| 1gpk | A | O | 0.47 | 0.29 | 0.83 | 0.72 | 0.94 |
| 1hq2 | A | O | 0.40 | 0.23 | 0.88 | 0.30 | 1.00 |
| 1hww | A | P | 0.00 | 0.00 | 0.00 | 0.00 | 0.00 |
| 1ia1 | A | O | 0.54 | 0.47 | 0.70 | 0.80 | 0.79 |
| 1ke5 | A | O | 0.14 | 0.18 | 0.17 | 0.78 | 0.13 |
| 1l7f | A | O | 0.30 | 0.28 | 0.38 | 0.20 | 0.17 |
| 1meh | A | O | 0.46 | 0.60 | 0.38 | 0.67 | 0.30 |
| 1n2v | A | O | 0.51 | 0.31 | 0.89 | 0.72 | 0.87 |
| 1nav | A | O | 0.48 | 0.45 | 0.60 | 1.00 | 0.87 |
| 1opk | A | O | 0.07 | 0.12 | 0.07 | 0.00 | 0.00 |
| 1oq5 | A | O | 0.58 | 0.40 | 0.91 | 0.65 | 0.88 |
| 1owe | A | O | 0.29 | 0.30 | 0.33 | 0.33 | 0.27 |
| 1p2y | A | O | 0.00 | 0.00 | 0.00 | 0.00 | 0.00 |
| 1r1h | A | O | 0.00 | 0.00 | 0.00 | 0.00 | 0.00 |
| 1r55 | A | O | 0.60 | 0.56 | 0.69 | 0.78 | 0.30 |
| 1r58 | A | O | 0.46 | 0.38 | 0.60 | 0.86 | 0.39 |
| 1r9o | A | O | 0.27 | 0.14 | 0.62 | 0.15 | 0.39 |
| 1s19 | A | O | 0.52 | 0.33 | 0.92 | 0.98 | 0.97 |
| 1s3v | A | O | 0.57 | 0.53 | 0.69 | 0.89 | 0.48 |
| 1sj0 | A | O | 0.41 | 0.50 | 0.38 | 1.00 | 0.27 |
| 1t40 | A | O | 0.45 | 0.41 | 0.56 | 0.61 | 0.79 |
| 1t46 | A | O | 0.51 | 0.57 | 0.50 | 0.86 | 0.43 |
| 1tow | A | O | 0.16 | 0.10 | 0.67 | 0.50 | 0.95 |
| 1uml | A | O | 0.59 | 0.41 | 0.92 | 0.72 | 0.79 |
| 1uou | A | O | -0.03 | 0.00 | 0.00 | 0.00 | 0.00 |
| 1v48 | A | P | 0.68 | 0.75 | 0.64 | 1.00 | 0.73 |
| 1v4s | A | O | 0.73 | 0.71 | 0.77 | 1.00 | 0.61 |
| 1vcj | A | O | 0.31 | 0.33 | 0.33 | 1.00 | 0.60 |
| 1x8x | A | P | 0.19 | 0.12 | 0.40 | 0.18 | 0.60 |
| 1xoz | A | O | 0.35 | 0.28 | 0.50 | 0.36 | 0.66 |
| 1y6b | A | O | 0.42 | 0.46 | 0.43 | 0.78 | 0.35 |
| 1yqy | A | O | 0.00 | 0.00 | 0.00 | 0.00 | 0.00 |
| 1yv3 | A | O | 0.00 | 0.00 | 0.00 | 0.00 | 0.00 |
| 1yvf | A | O | 0.00 | 0.00 | 0.00 | 0.00 | 0.00 |
| 1ywr | A | O | 0.05 | 0.09 | 0.08 | 0.40 | 0.14 |
| 1z95 | A | O | 0.23 | 0.25 | 0.13 | 0.00 | 0.00 |
| 2br1 | A | O | 0.10 | 0.12 | 0.17 | 0.25 | 0.07 |
| 2bsm | A | O | 0.43 | 0.40 | 0.57 | 0.67 | 0.22 |
| 1hwi | AB | O | 0.14 | 0.14 | 0.14 | 0.00 | 0.00 |
| 1gm8 | B | O | 0.00 | 0.00 | 0.00 | 0.00 | 0.00 |
| 1ig3 | B | O | 0.00 | 0.00 | 0.00 | 0.00 | 0.00 |
| 1lpz | B | O | 0.50 | 0.57 | 0.50 | 0.78 | 0.38 |
| 1mzc | B | O | 0.07 | 0.08 | 0.10 | 0.00 | 0.00 |
| 1p62 | B | O | 0.48 | 0.36 | 0.73 | 0.88 | 1.00 |
| 1oyt | H | O | 0.48 | 0.55 | 0.46 | 0.82 | 0.57 |
| 1ygc | H | O | 0.46 | 0.55 | 0.43 | 1.00 | 0.42 |

***Table S2. Prediction results of FPOCKET for protein structures in CCDC/Asterx dataset***

| ***Entry*** | | ***Site prediction*** | | ***Ligand space distribution*** | |
| --- | --- | --- | --- | --- | --- |
|  |  |  |  |  |  |
| ***PDB*** | ***Chain*** | ***Precision*** | ***Recall*** | ***Precision*** | ***Recall*** |
| 1g9v | A | 0.45 | 0.78 | 0.95 | 0.62 |
| 1gkc | A | 0.44 | 0.91 | 0.54 | 0.90 |
| 1hnn | A | 0.42 | 1.00 | 0.59 | 1.00 |
| 1hp0 | A | 0.68 | 1.00 | 0.59 | 1.00 |
| 1hvy | A | 0.25 | 0.90 | 0.38 | 0.71 |
| 1j3j | A | 0.52 | 1.00 | 0.58 | 1.00 |
| 1jd0 | A | 0.00 | 0.00 | 0.00 | 0.00 |
| 1jje | A | 0.44 | 1.00 | 0.68 | 1.00 |
| 1jla | A | 0.36 | 1.00 | 0.81 | 1.00 |
| 1k3u | A | 0.46 | 0.93 | 0.72 | 1.00 |
| 1kzk | A | 0.00 | 0.00 | 0.00 | 0.00 |
| 1l2s | A | 0.15 | 1.00 | 0.33 | 1.00 |
| 1lrh | A | 0.32 | 0.80 | 0.04 | 1.00 |
| 1m2z | A | 0.39 | 1.00 | 0.84 | 1.00 |
| 1mmv | A | 0.13 | 0.90 | 0.16 | 0.93 |
| 1n1m | A | 0.00 | 0.00 | 0.00 | 0.00 |
| 1n2j | A | 0.09 | 1.00 | 0.16 | 0.83 |
| 1n46 | A | 0.66 | 1.00 | 0.98 | 1.00 |
| 1of1 | A | 0.36 | 1.00 | 0.51 | 0.94 |
| 1of6 | A | 0.11 | 1.00 | 0.02 | 1.00 |
| 1pmm | A | 0.00 | 0.00 | 0.00 | 0.00 |
| 1q1g | A | 0.09 | 1.00 | 0.45 | 0.95 |
| 1q4g | A | 0.60 | 0.87 | 0.98 | 0.91 |
| 1q41 | A | 0.30 | 1.00 | 0.84 | 1.00 |
| 1sg0 | A | 0.80 | 0.42 | 0.96 | 0.41 |
| 1sq5 | A | 0.30 | 0.92 | 0.49 | 1.00 |
| 1sqn | A | 0.34 | 1.00 | 0.92 | 1.00 |
| 1t9b | A | 0.65 | 0.93 | 0.88 | 1.00 |
| 1tt1 | A | 0.32 | 1.00 | 0.58 | 0.93 |
| 1tz8 | A | 0.00 | 0.00 | 0.00 | 0.00 |
| 1u1c | A | 0.24 | 0.85 | 0.69 | 1.00 |
| 1u4d | A | 0.32 | 1.00 | 0.64 | 1.00 |
| 1unl | A | 0.39 | 0.64 | 0.45 | 0.50 |
| 1v0p | A | 0.38 | 1.00 | 0.64 | 0.90 |
| 1w1p | A | 0.27 | 1.00 | 0.25 | 0.91 |
| 1w2g | A | 0.36 | 1.00 | 0.84 | 0.94 |
| 1xm6 | A | 0.24 | 1.00 | 0.53 | 1.00 |
| 1xoq | A | 0.05 | 0.50 | 0.12 | 0.80 |
| 2bm2 | A | 0.47 | 1.00 | 0.54 | 0.45 |
| 1gpk | A | 0.33 | 1.00 | 0.51 | 0.59 |
| 1hq2 | A | 0.06 | 1.00 | 0.19 | 1.00 |
| 1hww | A | 0.00 | 0.00 | 0.00 | 0.00 |
| 1ia1 | A | 0.75 | 0.95 | 0.94 | 1.00 |
| 1ke5 | A | 0.32 | 1.00 | 0.37 | 1.00 |
| 1l7f | A | 0.93 | 0.92 | 0.91 | 1.00 |
| 1meh | A | 0.32 | 1.00 | 0.44 | 1.00 |
| 1n2v | A | 0.33 | 1.00 | 0.82 | 1.00 |
| 1nav | A | 0.46 | 1.00 | 0.80 | 1.00 |
| 1opk | A | 0.02 | 0.07 | 0.00 | 0.00 |
| 1oq5 | A | 0.00 | 0.00 | 0.00 | 0.00 |
| 1owe | A | 0.25 | 1.00 | 0.18 | 1.00 |
| 1p2y | A | 0.36 | 1.00 | 0.58 | 0.92 |
| 1r1h | A | 0.38 | 1.00 | 0.01 | 1.00 |
| 1r55 | A | 0.05 | 0.33 | 0.68 | 0.85 |
| 1r58 | A | 0.38 | 1.00 | 0.71 | 1.00 |
| 1r9o | A | 0.15 | 1.00 | 0.03 | 0.23 |
| 1s19 | A | 0.38 | 1.00 | 0.85 | 1.00 |
| 1s3v | A | 0.38 | 1.00 | 0.58 | 1.00 |
| 1sj0 | A | 0.35 | 1.00 | 0.71 | 1.00 |
| 1t40 | A | 0.56 | 0.72 | 0.58 | 1.00 |
| 1t46 | A | 0.43 | 1.00 | 0.85 | 1.00 |
| 1tow | A | 0.22 | 0.83 | 0.95 | 1.00 |
| 1uml | A | 0.36 | 1.00 | 0.81 | 1.00 |
| 1uou | A | 0.13 | 0.33 | 0.00 | 0.00 |
| 1v48 | A | 0.73 | 0.78 | 0.00 | 0.00 |
| 1v4s | A | 0.31 | 1.00 | 0.28 | 1.00 |
| 1vcj | A | 0.00 | 0.00 | 0.00 | 0.00 |
| 1x8x | A | 0.23 | 1.00 | 0.48 | 1.00 |
| 1xoz | A | 0.24 | 1.00 | 0.49 | 1.00 |
| 1y6b | A | 0.00 | 0.00 | 0.00 | 0.00 |
| 1yqy | A | 0.17 | 1.00 | 0.25 | 0.71 |
| 1yv3 | A | 0.13 | 1.00 | 0.22 | 1.00 |
| 1yvf | A | 0.24 | 0.70 | 0.35 | 0.55 |
| 1ywr | A | 0.33 | 1.00 | 0.52 | 1.00 |
| 1z95 | A | 0.51 | 1.00 | 0.89 | 1.00 |
| 2br1 | A | 0.44 | 0.91 | 0.62 | 0.82 |
| 2bsm | A | 0.60 | 1.00 | 0.93 | 1.00 |
| 1hwi | A | 0.00 | 0.00 | 0.00 | 0.00 |
| 1gm8 | B | 0.20 | 0.80 | 0.34 | 0.44 |
| 1ig3 | B | 0.11 | 0.33 | 0.00 | 0.00 |
| 1lpz | B | 0.44 | 0.81 | 0.71 | 0.69 |
| 1mzc | B | 0.00 | 0.00 | 0.00 | 0.00 |
| 1p62 | B | 0.83 | 1.00 | 0.12 | 1.00 |
| 1oyt | H | 0.29 | 1.00 | 0.58 | 0.96 |
| 1ygc | H | 0.23 | 0.53 | 0.89 | 0.92 |

***Table S3. Prediction results of CAVIAR for protein structures in CCDC/Asterx dataset***

| ***Entry*** | | ***Ligand space distribution*** | |
| --- | --- | --- | --- |
|  |  |  |  |
| ***PDB*** | ***Chain*** | ***Precision*** | ***Recall*** |
| 1g9v | A | 0.99 | 0.81 |
| 1gkc | A | 0.71 | 0.85 |
| 1hnn | A | 0.66 | 1.00 |
| 1hp0 | A | 0.80 | 0.95 |
| 1hvy | A | 0.72 | 0.98 |
| 1j3j | A | 0.59 | 1.00 |
| 1jd0 | A | 0.01 | 0.29 |
| 1jje | A | 0.96 | 1.00 |
| 1jla | A | 0.97 | 1.00 |
| 1k3u | A | 0.62 | 1.00 |
| 1kzk | A | 0.00 | 0.00 |
| 1l2s | A | 0.36 | 1.00 |
| 1lrh | A | 0.92 | 0.93 |
| 1m2z | A | 1.00 | 1.00 |
| 1mmv | A | 0.26 | 0.93 |
| 1n1m | A | 0.00 | 0.00 |
| 1n2j | A | 0.24 | 0.90 |
| 1n46 | A | 1.00 | 1.00 |
| 1of1 | A | 1.00 | 0.94 |
| 1of6 | A | 0.03 | 1.00 |
| 1pmm | A | 0.00 | 0.00 |
| 1q1g | A | 0.51 | 0.95 |
| 1q4g | A | 0.23 | 0.95 |
| 1q41 | A | 0.76 | 1.00 |
| 1sg0 | A | 0.97 | 0.33 |
| 1sq5 | A | 0.48 | 0.93 |
| 1sqn | A | 1.00 | 1.00 |
| 1t9b | A | 0.72 | 1.00 |
| 1tt1 | A | 0.73 | 0.93 |
| 1tz8 | A | 0.00 | 0.00 |
| 1u1c | A | 0.89 | 1.00 |
| 1u4d | A | 0.87 | 1.00 |
| 1unl | A | 0.94 | 1.00 |
| 1v0p | A | 0.93 | 0.96 |
| 1w1p | A | 0.16 | 0.91 |
| 1w2g | A | 0.95 | 0.94 |
| 1xm6 | A | 0.53 | 1.00 |
| 1xoq | A | 0.01 | 0.40 |
| 2bm2 | A | 1.00 | 0.48 |
| 1gpk | A | 0.22 | 1.00 |
| 1hq2 | A | 0.45 | 1.00 |
| 1hww | A | 0.43 | 1.00 |
| 1ia1 | A | 0.85 | 1.00 |
| 1ke5 | A | 0.03 | 0.30 |
| 1l7f | A | 0.59 | 1.00 |
| 1meh | A | 0.43 | 1.00 |
| 1n2v | A | 0.37 | 1.00 |
| 1nav | A | 0.36 | 1.00 |
| 1opk | A | 0.14 | 1.00 |
| 1oq5 | A | 0.59 | 0.54 |
| 1owe | A | 0.58 | 0.81 |
| 1p2y | A | 0.66 | 0.97 |
| 1r1h | A | 0.13 | 0.62 |
| 1r55 | A | 0.89 | 0.85 |
| 1r58 | A | 0.51 | 1.00 |
| 1r9o | A | 0.21 | 1.00 |
| 1s19 | A | 0.85 | 1.00 |
| 1s3v | A | 0.53 | 1.00 |
| 1sj0 | A | 0.59 | 1.00 |
| 1t40 | A | 0.47 | 1.00 |
| 1t46 | A | 0.62 | 1.00 |
| 1tow | A | 0.77 | 1.00 |
| 1uml | A | 0.58 | 1.00 |
| 1uou | A | 0.27 | 1.00 |
| 1v48 | A | 0.47 | 1.00 |
| 1v4s | A | 0.42 | 1.00 |
| 1vcj | A | 0.41 | 1.00 |
| 1x8x | A | 0.19 | 1.00 |
| 1xoz | A | 0.36 | 1.00 |
| 1y6b | A | 0.45 | 0.97 |
| 1yqy | A | 0.16 | 0.71 |
| 1yv3 | A | 0.19 | 1.00 |
| 1yvf | A | 0.06 | 0.85 |
| 1ywr | A | 0.35 | 1.00 |
| 1z95 | A | 0.47 | 1.00 |
| 2br1 | A | 0.60 | 0.97 |
| 2bsm | A | 1.00 | 1.00 |
| 1hwi | A | 0.00 | 0.00 |
| 1gm8 | B | 0.00 | 0.00 |
| 1ig3 | B | 0.00 | 0.00 |
| 1lpz | B | 1.00 | 0.75 |
| 1mzc | B | 0.00 | 0.00 |
| 1p62 | B | 0.46 | 1.00 |
| 1oyt | H | 0.77 | 0.96 |
| 1ygc | H | 0.65 | 0.83 |

***Table S4. Selection of optimal pockets for protein structures in CCDC/Asterx dataset by combining DeepbindPoc***

| **Entry** | | | **Site prediction** | | | **Ligand space distribution** | |
| --- | --- | --- | --- | --- | --- | --- | --- |
| **PDB** | **Chain** | **Cluster** | **MCC** | **Precision** | **Recall** | **Precision** | **Recall** |
| 1g9v | A | O | 0.45 | 0.32 | 0.86 | 0.76 | 0.98 |
| 1gkc | A | P | 0.27 | 0.33 | 0.25 | 0.00 | 0.00 |
| 1hnn | A | Q | -0.03 | 0.00 | 0.00 | 0.00 | 0.00 |
| 1hp0 | A | O | 0.69 | 0.50 | 1.00 | 0.93 | 1.00 |
| 1hvy | A | O | 0.38 | 0.21 | 0.82 | 0.41 | 0.81 |
| 1j3j | A | Q | -0.01 | 0.00 | 0.00 | 0.00 | 0.00 |
| 1jd0 | A | Q | -0.01 | 0.00 | 0.00 | 0.00 | 0.00 |
| 1jje | A | O | 0.49 | 0.33 | 0.82 | 0.56 | 1.00 |
| 1jla | A | O | 0.22 | 0.13 | 0.44 | 0.14 | 0.33 |
| 1k3u | A | Q | -0.05 | 0.00 | 0.00 | 0.00 | 0.00 |
| 1kzk | A | O | 0.49 | 0.55 | 0.55 | 0.45 | 0.44 |
| 1l2s | A | Q | -0.01 | 0.00 | 0.00 | 0.00 | 0.00 |
| 1lrh | A | Q | -0.01 | 0.00 | 0.00 | 0.00 | 0.00 |
| 1m2z | A | O | 0.17 | 0.22 | 0.18 | 0.00 | 0.00 |
| 1mmv | A | R | -0.00 | 0.00 | 0.00 | 0.00 | 0.00 |
| 1n1m | A | Q | -0.00 | 0.00 | 0.00 | 0.00 | 0.00 |
| 1n2j | A | P | 0.19 | 0.33 | 0.12 | 0.00 | 0.00 |
| 1n46 | A | O | 0.01 | 0.08 | 0.06 | 0.00 | 0.00 |
| 1of1 | A | O | 0.82 | 0.78 | 0.88 | 1.00 | 1.00 |
| 1of6 | A | O | 0.66 | 0.71 | 0.62 | 0.40 | 0.62 |
| 1pmm | A | R | -0.02 | 0.00 | 0.00 | 0.00 | 0.00 |
| 1q1g | A | O | 0.44 | 0.31 | 0.73 | 0.58 | 1.00 |
| 1q4g | A | S | -0.00 | 0.00 | 0.00 | 0.00 | 0.00 |
| 1q41 | A | O | 0.06 | 0.07 | 0.12 | 0.50 | 0.29 |
| 1sg0 | AB | O | 0.45 | 0.29 | 0.75 | 0.00 | 0.00 |
| 1sq5 | A | O | 0.54 | 0.58 | 0.54 | 0.29 | 0.48 |
| 1sqn | A | O | 0.21 | 0.17 | 0.38 | 0.00 | 0.00 |
| 1t9b | A | O | 0.18 | 0.08 | 0.50 | 0.00 | 0.00 |
| 1tt1 | A | Q | -0.04 | 0.00 | 0.00 | 0.00 | 0.00 |
| 1tz8 | AB | O | 0.72 | 0.47 | 1.00 | 0.22 | 0.50 |
| 1u1c | A | P | -0.02 | 0.00 | 0.00 | 0.00 | 0.00 |
| 1u4d | A | O | 0.47 | 0.28 | 0.89 | 0.47 | 0.94 |
| 1unl | A | Q | -0.01 | 0.00 | 0.00 | 0.00 | 0.00 |
| 1v0p | A | Q | -0.03 | 0.00 | 0.00 | 0.00 | 0.00 |
| 1w1p | A | S | 0.23 | 0.33 | 0.17 | 0.00 | 0.00 |
| 1w2g | A | O | 0.29 | 0.11 | 1.00 | 0.07 | 1.00 |
| 1xm6 | A | O | 0.47 | 0.31 | 0.80 | 0.58 | 1.00 |
| 1xoq | A | O | 0.59 | 0.43 | 0.87 | 0.56 | 1.00 |
| 2bm2 | A | P | 0.05 | 0.09 | 0.09 | 0.00 | 0.00 |
| 1gpk | A | P | -0.01 | 0.00 | 0.00 | 0.00 | 0.00 |
| 1hq2 | A | O | 0.51 | 0.34 | 0.92 | 0.69 | 1.00 |
| 1hww | A | S | 0.15 | 0.25 | 0.09 | 0.00 | 0.00 |
| 1ia1 | A | P | 0.36 | 0.53 | 0.33 | 0.24 | 0.27 |
| 1ke5 | A | P | 0.26 | 0.33 | 0.25 | 0.33 | 0.13 |
| 1l7f | A | R | 0.49 | 0.50 | 0.50 | 0.00 | 0.00 |
| 1meh | A | P | 0.33 | 0.43 | 0.27 | 0.00 | 0.00 |
| 1n2v | A | O | 0.50 | 0.30 | 0.89 | 0.62 | 1.00 |
| 1nav | A | O | 0.48 | 0.45 | 0.60 | 1.00 | 0.87 |
| 1opk | A | O | 0.07 | 0.05 | 0.17 | 0.00 | 0.00 |
| 1oq5 | A | O | 0.60 | 0.39 | 1.00 | 0.67 | 1.00 |
| 1owe | A | O | 0.50 | 0.27 | 1.00 | 0.29 | 1.00 |
| 1p2y | A | P | -0.01 | 0.00 | 0.00 | 0.00 | 0.00 |
| 1r1h | A | R | -0.01 | 0.00 | 0.00 | 0.00 | 0.00 |
| 1r55 | A | Q | -0.02 | 0.00 | 0.00 | 0.00 | 0.00 |
| 1r58 | A | O | 0.52 | 0.41 | 0.70 | 0.83 | 0.70 |
| 1r9o | A | P | -0.01 | 0.00 | 0.00 | 0.00 | 0.00 |
| 1s19 | A | Q | -0.05 | 0.00 | 0.00 | 0.00 | 0.00 |
| 1s3v | A | O | 0.57 | 0.53 | 0.69 | 0.89 | 0.48 |
| 1sj0 | A | Q | 0.11 | 0.25 | 0.08 | 0.00 | 0.00 |
| 1t40 | A | Q | 0.19 | 0.67 | 0.07 | 0.27 | 0.25 |
| 1t46 | A | O | 0.72 | 0.47 | 0.50 | 0.33 | 0.51 |
| 1tow | A | P | 0.30 | 0.33 | 0.33 | 0.00 | 0.00 |
| 1uml | A | P | -0.01 | 0.00 | 0.00 | 0.00 | 0.00 |
| 1uou | A | O | -0.03 | 0.00 | 0.00 | 0.00 | 0.00 |
| 1v48 | A | Q | -0.01 | 0.00 | 0.00 | 0.00 | 0.00 |
| 1v4s | A | P | -0.00 | 0.00 | 0.00 | 0.00 | 0.00 |
| 1vcj | A | P | -0.03 | 0.00 | 0.00 | 0.00 | 0.00 |
| 1x8x | A | O | -0.02 | 0.00 | 0.00 | 0.00 | 0.00 |
| 1xoz | A | O | 0.36 | 0.25 | 0.60 | 0.39 | 0.66 |
| 1y6b | A | O | 0.56 | 0.48 | 0.71 | 0.81 | 0.79 |
| 1yqy | A | O | -0.01 | 0.00 | 0.00 | 0.00 | 0.00 |
| 1yv3 | A | P | -0.00 | 0.00 | 0.00 | 0.00 | 0.00 |
| 1yvf | A | P | 0.31 | 0.10 | 1.00 | 0.16 | 1.00 |
| 1ywr | A | Q | -0.03 | 0.00 | 0.00 | 0.00 | 0.00 |
| 1z95 | A | P | 0.09 | 0.15 | 0.13 | 0.00 | 0.00 |
| 2br1 | A | O | 0.39 | 0.30 | 0.58 | 0.40 | 0.45 |
| 2bsm | A | O | 0.52 | 0.43 | 0.71 | 0.78 | 0.78 |
| 1hwi | AB | O | 0.26 | 0.25 | 0.29 | 0.00 | 0.00 |
| 1lpz | B | O | 0.60 | 0.62 | 0.62 | 0.87 | 0.88 |
| 1mzc | B | O | 0.07 | 0.08 | 0.10 | 0.00 | 0.00 |
| 1p62 | B | O | 0.48 | 0.36 | 0.73 | 0.88 | 1.00 |
| 1ig3 | B | P | -0.01 | 0.00 | 0.00 | 0.00 | 0.00 |
| 1gm8 | B | O | -0.02 | 0.00 | 0.00 | 0.00 | 0.00 |
| 1oyt | H | O | 0.51 | 0.43 | 0.69 | 0.73 | 0.97 |
| 1ygc | H | O | 0.47 | 0.50 | 0.50 | 0.96 | 0.89 |

***Figure S1. MDPA calculation results of SARS-COV-2***

***
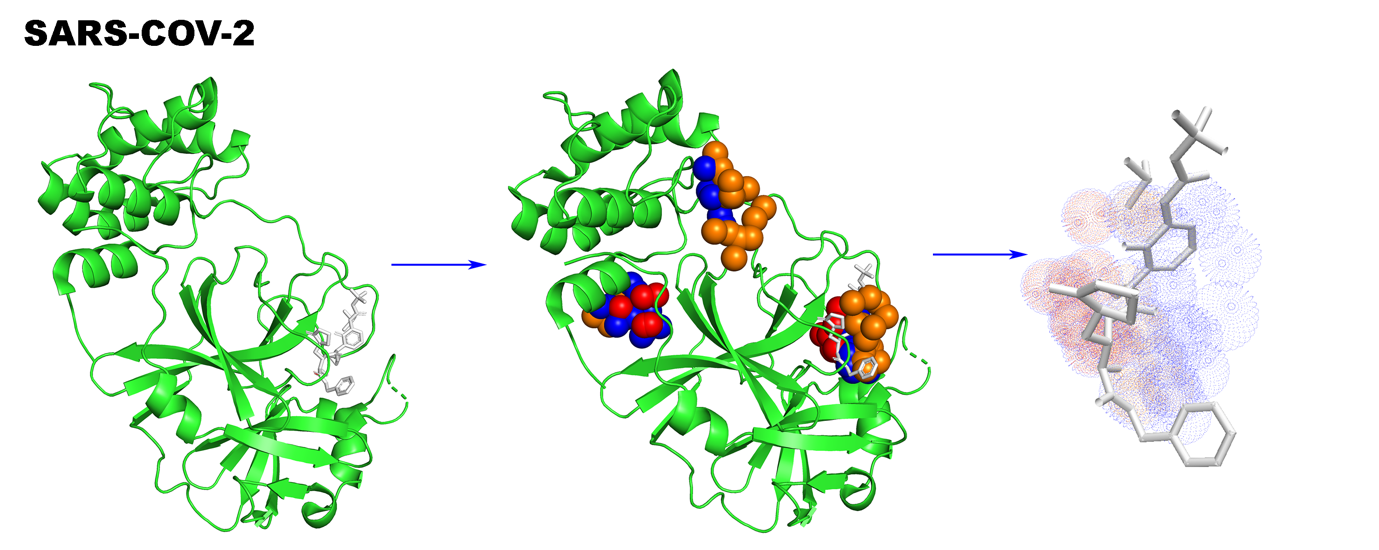
***
